# SNPs in cancer research and treatment

**DOI:** 10.1038/sj.bjc.6601574

**Published:** 2004-02-17

**Authors:** H C Erichsen, S J Chanock

**Affiliations:** 1 Section on Genomic Variation, Pediatric Oncology Branch, Center for Cancer Research, Advanced Technology Center, National Cancer Institute, National Institutes of Health, 8718 Grovemont Circle, Bethesda, MD 20892-4605, USA; 2 Core Genotyping Facility, National Cancer Institute, National Institutes of Health, Bethesda, MD, USA

**Keywords:** cancer, genomics, haplotype, single-nucleotide polymorphism

## Abstract

Genetic variation in the human genome is an emerging resource for studying cancer, a complex set of diseases characterised by both environmental and genetic contributions. The number of common germ-line variants is great, on the order of 10–15 million per person, and represents a remarkable opportunity to investigate the aetiology, interindividual differences in treatment response and outcomes of specific cancers. The study of genetic variation can elucidate critical determinants in environmental exposure and cancer, which could have future implications for preventive and early intervention strategies. However, we are in the initial stages of characterising the tools (i.e., the single-nucleotide polymorphism, SNP) to rigorously analyse the genetic contributions to complex diseases, such as cancer. If the promise of the genomic era is to be realised, we must integrate this information into new strategies for implementation in both public health measures and, most importantly, provision of individual cancer-related care.

## GENETIC VARIATION IN THE HUMAN GENOME

In the process of generating a draft sequence of the human genome, it has become clear that the extent of genetic variation is much larger than previously estimated (Lander *et al*, 2001; Venter *et al*, 2001). The most common sequence variation in the human genome is the stable substitution of a single base, the single-nucleotide polymorphism (SNP). By definition, a SNP has a minor allele frequency of greater than 1% in at least one population (Risch, 2000). Most SNPs are ‘silent’ and do not alter the function or expression of a gene. It makes sense to conceptually reserve the term ‘mutation’ for rare variants with a particularly high penetrance, usually associated with a detrimental phenotype, such as a classical monogenic disorder (e.g., sickle cell disease or haemophilia). For the purposes of this review, the focus will be on SNPs with low penetrance or no phenotypic effect.

The total number of SNPs in the human genome is estimated to be more than 10 million (Botstein and Risch, 2003) and the number of SNPs with a minor allele frequency over 10% is estimated to be perhaps as many as five million (Kruglyak and Nickerson, 2001). Single-nucleotide polymorphisms are distributed throughout the human genome, at an estimated overall frequency of at least on in every 1000 base pair (bp) (Carlson *et al*, 2003), but with marked regional differences. Single-nucleotide polymorphisms arise because of point mutations that are selectively maintained in populations. Single-nucleotide polymorphism frequencies are determined by: (1) the amount of time since the mutation occurred; (2) evolutionary pressure on biologically significant variants and those linked to the functional variant; (3) random genetic drift and (4) bottleneck events.

**Figure 1:**
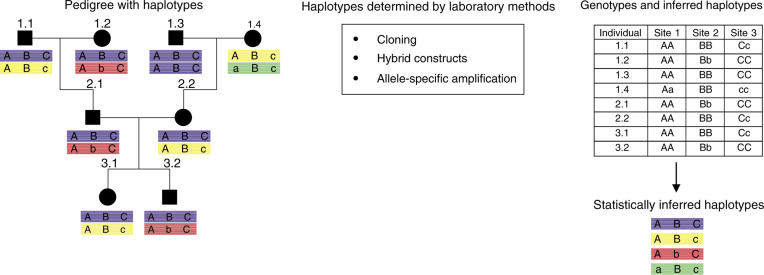
Determination of haplotypes in unrelated and related subjects. Haplotype structure is determined in pedigrees on the basis of genotype analysis at three different sites of a chromosomal region. The major and minor alleles are represented by A/a, B/b and C/c, respectively. The haplotypes can be deduced from the data in the table, and follow classical Mendelian inheritance. Haplotypes can be inferred in unrelated subjects by applying statistical algorithms to estimate haplotypes, based on genotype data. However, this will not determine a haplotype unequivocally, rather giving a haplotype with a statistical probability of being correct. Although this approach is most widely used because of its cost effectiveness, other methods exist that will determine haplotypes conclusively. On occasion, one can isolate DNA clones from a single chromosome for direct sequence analysis or selectively amplify using allele-specific oligonucleotide primers.

Single-nucleotide polymorphisms in the same chromosomal region are not inherited randomly, but as combinations of alleles, which form haplotype blocks. It appears that the genome is organised into distinct blocks of linkage disequilibrium (LD), intercepted by regions in which LD breaks down rapidly (Bonnen *et al*, 2002; Sabeti *et al*, 2002). Thus, the complexity of analysing SNPs in a gene or locus can be reduced by the analysis of markers inherited on a haplotype. Practically, haplotypes can be inferred by LD analysis of a region in unrelated subjects, or characterised molecularly in family pedigrees (see Figure 1

). It is notable that both the frequency of SNPs and the extent of LD may vary significantly between populations. In addition, there are many population private variants (Carlson *et al*, 2003).

## SNPS AND PHENOTYPE

As the age of genomics proceeds forward in search of genetic variants (i.e. SNPs) that influence disease susceptibility and outcome, a great effort has been directed at picking SNPs for study. The promise of utilising biallelic SNPs for whole genome linkage studies, though very exciting in theory, is still some time off in the future because of issues related to the prohibitive cost and impracticality of genotyping the required thousands of SNPs, not to mention developing the databases and analytical tools needed to interpret the data (Kruglyak, 1999). Still, we can count on this approach in the future, but for now, because of limited resources and analytical tools, most investigators will continue to utilise a strategy that investigates specific genes, known as the candidate gene approach.

The candidate gene approach examines SNPs, chosen from genes that ‘make sense’. In other words, they fit a plausible understanding of the biology. Single-nucleotide polymorphisms can also be chosen from a region previously identified by linkage study or microarray expression analysis. Intense effort has focused on SNPs that alter protein function or gene expression. A hierarchy for predicting a possible phenotypic expression for a SNP has been proposed (Risch, 2000). It has been estimated that there are perhaps 50 000–250 000 SNPs which confer a biological effect, most of which are distributed in and around the 30 000 genes (Risch, 2001). Prediction of a biological effect is probably more complicated than once argued; for instance, a synonymous SNP, which does not alter the amino-acid sequence has been shown to influence the stability of the *DRD2* transcript, resulting in alterated expression (Duan *et al*, 2003).

Since the majority of SNPs do not confer a phenotypic alteration, but lie on ancestral haplotypes, a critical distinction must be made between SNPs as genetic markers and causal factors associated with a phenotypic effect. To date, the literature has focused on SNPs that have a predicted or demonstrated functional effect. A candidate gene approach that includes haplotype-tagging SNPs investigates genetic variation across the gene or locus. Once a haplotype has been confirmed as a marker for a phenotype, it is necessary to analyse the component SNPs to determine the causal variants. In some cases, it might be necessary to study additional SNPs, to better define the haplotype structure in search of causal variants. Attempts to by-pass this and pick only ‘functionally’ important SNPs limits the opportunity to ‘mark’ a gene or region. Moreover, there is a certain amount of hubris to continue to expect that picking individual SNPs is an effective strategy to identify alterations in genes that could contribute to complex diseases, such as cancer. No doubt, preclinical studies will suggest which variants to study, but the technical and bioinformatics advances have already generated a rich resource for study, which includes many genes and SNPs, of which little is known.

## STUDY DESIGN

Traditionally, two different methods have been applied to the study of genetic variants in human disease. In SNP and haplotype studies, the measure of effect is a change in risk, that is, attributable risk. The results indicate a nonrandom distribution of genetic variants between cases and controls. In general, a SNP finding is neither necessary nor sufficient for the complex disease under study. Firstly, in highly penetrant monogenic disorders, family linkage analysis has been successful in identifying many rare genetic disorders. So far, more than 1200 disease-causing genes have been identified (http://www.ncbi.nlm.nih.gov:80 /entrez/query.fcgi?db=OMIM). For complex diseases, such as most cancers, the paucity of family pedigrees has limited the success of this strategy, especially in search of moderate or low penetrance genes (Risch, 2001). Even in instances in which rare germ-line mutations in *TP53* or *VHL* genes have been mapped, penetrance is not complete (i.e., the presence of a mutation does not necessarily lead to cancer); this observation suggests that additional modifying genes as well as environmental factors contribute to cancer even in the high-risk setting. In studies of subjects with a monogenic disorder (i.e., cystic fibrosis or chronic granulomatous disease), it has been possible to identify modifying variants that influence the risk for well-defined phenotypes (Foster *et al*, 1998; Garred *et al*, 1999).

The second strategy, which is more commonly employed, is the genetic association study in unrelated subjects. Collection of larger data sets of cases and controls permits investigation of SNPs and haplotypes, which confer a moderate or low effect. Until recently, the primary approach utilised has been the case–control design, but, recently, many have advocated turning to population-based cohort studies. Traditionally, case–control studies have been designed to detect susceptibility loci and, rarely, protective alleles (Risch and Merikangas, 1996; Risch, 2000).

## ANALYTICAL ISSUES IN SNP STUDIES

Estimation of LD and construction of haplotypes represents a powerful tool for conducting association studies (Gabriel *et al*, 2002). Even if the extent of LD and the number of haplotypes vary across the genome and between populations, it appears that there is generally low haplotype diversity for each locus, with some differences observed in separate populations (Daly *et al*, 2001). Consequently, association studies can genotype a limited set of SNPs that contribute to common haplotypes, with haplotype-tagging SNPs (ht-SNPs) (Stram, 2003). Resequence analysis is required to capture sufficient diversity in populations of unrelated subjects, which can be analysed to infer haplotype structure across the locus (Bonnen *et al*, 2002). Analysis with ht-SNPs saves money and DNA, but requires new analytical tools to estimate the effect of individual haplotypes on both the main effect (i.e., susceptibility or outcome) and gene–gene interactions. Already several studies have demonstrated the utility of haplotype analysis; for instance, an association has been reported between haplotypes on chromosome 19 and basal cell carcinoma; similarly, haplotypes of the interleukin-4 promoter are associated with chronic disseminated candidiasis, a life-threatening infection in patients with acute leukaemia (Yin *et al*, 2002; Choi *et al*, 2003).

Association studies have been plagued by an inability to consistently yield reproducible results. Several factors contribute to this conundrum and are hotly debated among experts in the field. Since replication is critical for acceptance of the causal association between a SNP or haplotype and an outcome, it has been argued that false-positive associations are better tolerated than false negatives. Thus far, the problem has been that there have been too many false positives. Some have argued that the execution of a large number of tests, by definition, contributes to the high rate of false positives, but others argue that the variables are not independent, certainly not when considering haplotypes (Colhoun *et al*, 2003; Lohmueller *et al*, 2003). Another factor for lacking reproducibility is inadequately powered studies, both in the initial and follow-up studies. Admixture of populations has been advanced as a confounding factor, though the effect has been demonstrated to be less than anticipated in published examples (Wacholder *et al*, 2000). Still, genetic drift coupled with the rate and genomic distribution of recombination and mutation events in populations under different pressures will affect LD and, thus, the potential to discover a given association (Frisse *et al*, 2001; Colhoun *et al*, 2003; Lohmueller *et al*, 2003). Currently, alternative remedies have been advanced that address novel statistical analysis, Bayesian approaches and meta-analyses of published studies. It is anticipated that current analytical tools will rapidly evolve in response to revisitation of data sets.

## SNPS IN CANCER RESEARCH

Genetic association studies with SNPs targeting cancer can be divided into two broad categories, investigation of susceptibility and of outcomes (see Table 1

**Table 1:** Examples of genes and associations

**Gene**	**Association**
*Cancer susceptibility*	
Myeloperoxidase, MPO	Lung cancer
*N*-acetyltransferase 1, NAT1	Bladder cancer
*N*-acetyltransferase, NAT2	Bladder/colon cancer
*Cancer outcome*	
CYP3A4	Prostate cancer
*Pharmacogenomics*	
Thiopurine *S*-methyltransferase, *TPMT*	Haematological toxicity

). The latter seeks to determine prognostic information for survival, complications or response to pharmacological intervention (i.e., pharmacogenomics). To date, nearly all published studies have examined at most a few SNPs or genes, and on a rare occasion variants within genes of a pathway or related biological process, such as DNA repair enzymes (i.e., *XRCC1* and *XRCC3*) or xenobiotic metabolism genes (i.e., *NAT1* and *NAT2*). Though early in the study of SNPs and cancer, technical and bioinformatics advances make it possible to dramatically increase the number of genes applied to a study. It is important to bear in mind that SNP studies require replication prior to acceptance, and certainly before clinical implementation. Therefore, it is critical to consider the current published literature as very preliminary analysis of what will certainly be a complex process.

### Susceptibility to cancer

The aetiology of a specific cancer is probably associated with a set of genetic variants, many of which could adversely interact with environmental factors. So far, the initial studies of single genes have established a paradigm that will eventually examine gene–gene interactions. Studies in lung cancer have interrogated genes important for tobacco metabolism and nicotine addiction. The approach focuses on the interaction of a strong environmental carcinogen, and seeks to identify genetic variants that confer susceptibility or protection from tobacco smoke. In this regard, the gene–environment interaction represents a stress on the host, and perhaps intensifies the phenotypic effect of the ‘causal’ SNP. For instance, the myeloperoxidase gene, *MPO*, has been extensively studied, yielding moderately reproducible data in studies with Caucasians. The functional consequence of the G to A transition at −463 of the proximal promoter leads to reduced *MPO* mRNA expression. Individuals homozygous for the A allele have a significantly lower risk for developing lung cancer compared to individuals with two G alleles (London *et al*, 1997; Cascorbi *et al*, 2000; Le Marchand *et al*, 2000).

It is notable that, in one context, the SNP or haplotype can be protective, whereas, in another, confer increased susceptibility. For example, one of the strongest cases can be made for the association of polymorphisms in *N*-acetyltransferase 2 (*NAT2*) with bladder and colon cancer. *NAT1* and *NAT2* encode enzymes that are important for biotransformation of aromatic and heterocyclic amines, known carcinogens. The risk of developing urinary bladder cancer is particularly high in the slowest NAT2 acetylator phenotype, and is exacerbated by the history of smoking. On the other hand, for heterocyclic amine-related colon cancer, *NAT2* rapid acetylator phenotype confers a higher risk (Golka *et al*, 2002; Hein, 2002).

### Outcome and SNPs

Variants can be associated with outcome and, thus, could be applied to clinical decision making. For instance, genetic variants could alter the risk for metastatic or aggressive tumour. To date, few studies have unequivocally shown the importance of germ-line variants as prognostic markers, but, since a tiny percentage of the known genes have been adequately studied, investigation of SNPs remains active. So far, preliminary results suggest that SNPs in *CYP3A4* are associated with long-term outcome in prostate cancer. The promoter SNP A-290G in *CYP3A4*, a gene involved in the oxidation of testosterone to 2B-, 6B, or 15B-hydroxytestosterone, appears to be associated with the severity of disease, as measured by TNM stage and Gleason grade. The effect is stronger in older men without a family history of prostate cancer (Rebbeck *et al*, 1998; Paris *et al*, 1999).

Pharmacogenomics is the study of the inherited basis of interindividual differences in drug response. It has been estimated that inherited differences account for inter individual variation observed in drug response (Kalow *et al*, 1998). There have been two parallel approaches in pharmacogenomics. One approach has been the search for genetic variants that are associated with severe adverse effects, which, in turn, can be used to screen for individuals who should not receive the drug in question. The second approach has focused on identifying markers that predict drug efficacy. The former has been widely embraced by the commercial sector, whereas the latter has been more tepidly pursued. Nevertheless, the promise of pharmacogenomics is that it could lead to tailored drug therapy, which some have dubbed as ‘individualised medicine.’ The harsh reality is that the choice facing an individual will have to be made on the basis of risk assessment gathered from large, population-based studies.

Candidate genes have focused on drug metabolism, that is, uptake, activation, degradation and excretion, to identify SNPs that are associated with life-threatening adverse reactions. Armed with validated SNPs and haplotype markers that associate with adverse drug reactions, clinicians could perform screening tests and, if indicated, choose alternate therapy. However, application to clinical medicine will be daunting, since many SNPs will identify risk factors that have only moderate-to-low penetrance. In other words, the clinician will be faced with assessing the overall risk for adverse effects, which will have to be weighed against potential benefits as well as the availability of alternative therapies.

A seminal example of the importance of pharmacogenomics is the story of acute childhood leukaemia and variants in the thiopurine S-methyltransferase (*TPMT)* gene. The protein encoded by the *TPMT* gene is a catalyst for S-methylation of thiopurines, commonly used in the treatment of haematopoietic malignancies and autoimmune disorders (e.g., azathiprine, 6-mercaptopurine and thioguanine). Thiopurine drugs are activated to thioguanine nucleotides, which are cytotoxic. Cytotoxicity can be intended (e.g., antileukaemia therapy), but if prolonged, life-threatening. It was discovered that several rare variants in the *TPMT* gene correlated with decreased activity; homozygous individuals suffered substantial haematopoietic toxicity (Krynetski *et al*, 1996). Recently, it has been reported that children with inactivating *TPMT* variants are at a greater risk for relapse, perhaps reflecting inadequate administration of thiopurines (Black *et al*, 1998). Pediatric leukaemia patients with *TPMT* variants, who receive cranial irradiation, have a greater likelihood for secondary brain tumours.

The search for markers useful for predicting drug efficacy has focused on the cytochrome *P*-450 system and, specifically, the *CYP2D6* gene. This gene contributes to the metabolism of many anticancer agents. Common SNPs in *CYP2D6* impair the activity of *CYP2D6* and perhaps alter the pharmacokinetics of anticancer drugs (Kroemer and Eichelbaum, 1995). In some circumstances, the impaired *CYP2D6* activity actually enhances the drug effect, but perhaps at the expense of enhanced toxicity.

## EPIGENETIC CHANGES AND GENETIC VARIATION

Genomic instability has been characterised in many human cancers and its signature is the pattern of sequence alterations, frequently resulting in allelic imbalance. Until recently, efforts to capture global patterns of genomic imbalance have employed microsatellites, but the utility of dense SNP markers has been demonstrated in proof-of-principle studies. Global patterns of genomic imbalance can be detected by allelotyping of cancers, and point to regions where allelic imbalance could contribute to cancer. Since loss of heterozygosity (LOH) within one or more chromosomal region is a common form of allelic imbalance, sites of LOH can be investigated for the presence of tumour-suppressor genes. Studies in bladder, lung and prostate cancers have discovered previously unknown allelic imbalances in multiple sites using SNPs for LOH analysis (Lindblad-Toh *et al*, 2000). It is likely that patterns of LOH, through SNP analysis, could have diagnostic and prognostic implications. Specific LOH pattern could be correlated with expression array profiles to identify causal variants.

## THE FUTURE OF SNPS AND HAPLOTYPES IN CANCER

The analysis of SNPs and haplotypes in cancer research has pleotropic implications for clinical and public health issues, as well as cancer biology. New findings can lead to targeted therapies for cancer intervention and prevention. The initial excitement of applying genetic variation to cancer has yielded to the realisation that a complex set of challenges lie ahead, which will tax the scientific, clinical and social adaptation of the new era of genomic medicine. Already, there are examples in pharmacogenomics and cancer susceptibility that have established the importance of this field, but it must be emphasised that they represent the beginning. The next decade could witness the investigation and implementation of genetic variation in the diagnosis, prevention and treatment of cancer.

The promise of SNP and haplotype analysis is that it will yield insights into exposure and cancer, and specifically lay the foundation for primary preventive strategies pertaining to lifestyle (i.e., alterations in diet, exercise and weight control) and chemoprevention. In turn, secondary prevention will address screening and public health measures designed to identify and intervene in high-risk individuals. The latter will become more complicated as we integrate information about pharmacogenomics, second malignancies and long-term complications. This is particularly complex because the consequences of one set of SNPs could have deleterious consequences for one outcome, but yet be advantageous for another.

A major challenge ahead is the development of more sophisticated analytical tools for handling the expanding data. We will need to address the complex issue of gene–gene interactions, particularly if profiles of SNPs are to be used in the clinical venue. At the same time, it is imperative to develop structures to include more individuals in population-based studies, yet preserve both confidentiality and individuality. In many respects, this is the most daunting challenge, to derive the information to interpret the significance of specific SNPs and haplotypes from population-based studies, yet apply the information to the individual. It could have a significant impact on the economics of medical practice.

In the end, the clinical application of what we learn from SNP studies will probably require a major shift in the paradigm of practice. Practitioners could make treatment decisions or give advice based on an assessment of risk. To begin to address the central issues of SNPs, haplotypes and cancer, detailed knowledge of the scope and pattern of genetic variation will be required, namely SNPs, haplotypes and linkage disequilibrium for genes and pathways critical to cancer in different populations.
